# Identification of tickborne pathogens in cattle and sheep ticks from Kyrgyzstan using next-generation sequencing

**DOI:** 10.1186/s13071-025-06919-4

**Published:** 2025-07-22

**Authors:** Ji Ye Seo, Jin Seo Park, Bekbolsun Aknazarov, Hee Il Lee

**Affiliations:** 1 Division of Vectors and Parasitic Diseases, Korea Disease Control and Prevention Agency, 187 Osongsaengmyeong 2-ro, Osong-eup, Heungdeok-gu, Cheongju, 28159 Chungbuk Republic of Korea; 2 Faculty of Veterinary Medicine, Kyrgyz National Agrarian University Named After K. I. Skryabin, Bishkek, 720005 Kyrgyzstan

**Keywords:** Tick, Tickborne pathogen, *Anaplasma*, *Ehrlichia*, Spotted fever group Rickettsiae, *Bartonella*, Next-generation sequencing

## Abstract

**Background:**

Various tickborne diseases and pathogens in livestock have been reported in Kyrgyzstan; however, comprehensive molecular analyses from ticks and their tickborne pathogen diversity in the region are lacking. This study aimed to identify tick species and bacterial pathogens infesting cattle and sheep across Kyrgyzstan using amplicon-based next-generation sequencing (NGS).

**Methods:**

In 2022, ticks were collected from livestock across seven provinces and subjected to molecular analyses. Genomic DNA was extracted from ticks for species identification based on cytochrome c oxidase I* (COI)* gene sequence analyses. Pathogens were screened using amplicon NGS targeting the V3–V4 region of the *16S rRNA* gene, followed by confirmation using polymerase chain reaction (PCR) and Sanger sequencing.

**Results:**

A total of 546 ticks belonging to two families, five genera, and 12 species were identified. The dominant species were *Dermacentor* spp. (30.2%), *Hyalomma marginatum* (17.2%), *Hyalomma scupense* (13.4%), and *Haemaphysalis punctata* (11.7%). Furthermore, 11.7% of ticks tested positive for bacterial pathogens, including spotted fever group Rickettsiae (8.6%), *Anaplasma* (2.7%),* Ehrlichia* (0.2%), and *Bartonella* (0.2%). *Coxiella burnetii* and *Francisella tularensis* were not detected.

**Conclusions:**

This is the first nationwide study on bacterial pathogens in ticks in Kyrgyzstan and the first reports of spotted fever group Rickettsiae and *Bartonella* in the country. These findings improve our understanding of tickborne disease epidemiology and highlight the utility of NGS as an efficient screening method for capturing pathogen diversity in arthropod vectors.

**Graphical Abstract:**

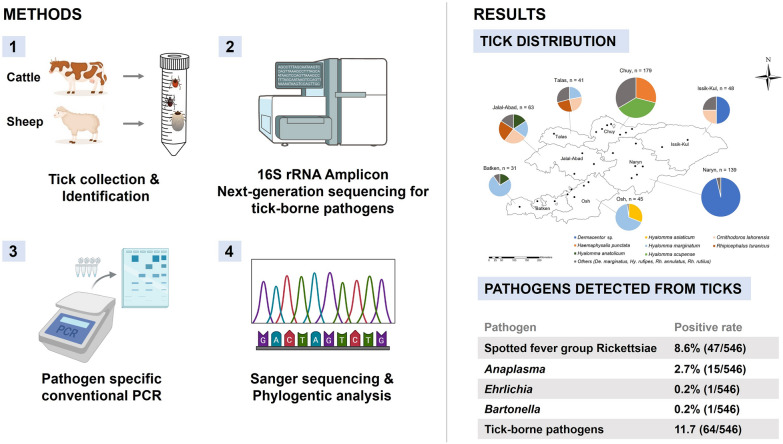

**Supplementary Information:**

The online version contains supplementary material available at 10.1186/s13071-025-06919-4.

## Background

Ticks (class Arachnida, subclass Acari, and order Ixodida) are highly adaptable hematophagous arthropods and are distributed worldwide. These ectoparasites act as vectors for a variety of tickborne pathogens, including bacteria, viruses, and protozoans. Among tick-transmitted bacterial pathogens, *Rickettsia*, *Anaplasma*, *Ehrlichia*, *Bartonella*, *Borrelia*, and *Francisella* pose significant risks to human and veterinary health [1, 2]. Furthermore, climate change may indirectly influence the distribution and abundance of ticks through changes in host availability, and tick abundance is closely linked to pathogen transmission rates [1]. Therefore, the continuous monitoring of ticks and their pathogens is essential to prevent and control tickborne diseases (TBDs) in humans and animals.

Kyrgyzstan, a Central Asian country situated between the Tian Shan Mountains and Pamir-Alay mountain range, shares borders with Kazakhstan, Tajikistan, Uzbekistan, and the Xinjiang Uygur Autonomous Region (XUAR) of China. With limited arable land and grasslands covering approximately 56% of the country, pastoralism has become a key economic activity. Cattle and sheep farming dominate the livestock sector, with the production of milk, meat, and wool being essential for both local consumption and export [3]. The climate of the Kyrgyzstan varies widely owing to its mountainous topography and continental location, ranging from subtropical in the Fergana Valley to temperate in the northern foothills and dry continental to polar in the Tien Shan. Additionally, the presence of Lake Issyk-Kul further contributes to localized variations, creating conditions from hypercontinental to nearly oceanic [4].

However, tickborne diseases (TBDs), such as tickborne encephalitis, Crimean–Congo hemorrhagic fever, and Siberian tick typhus, have been reported, with 111, 1, and 86 cases, respectively, documented between 2014 and 2023 [5]. Various tickborne pathogens, including *Anaplasma* spp., *Theileria* spp., and *Babesia* spp., have been detected in the blood of livestock. Furthermore, molecular detection methods have confirmed the presence of tickborne viruses (tickborne encephalitis virus) and bacteria (*Anaplasma* spp. and *Ehrlichia* spp.) in ticks from the region [6–10]. However, these studies were limited to predefined pathogens and may not fully capture the diversity of bacterial pathogens in the area.

Amplicon next-generation sequencing (NGS) is a powerful, highly targeted approach that amplifies the conserved regions of specific genes, such as the 16S ribosomal RNA gene, without the need for species-specific primers [11]. This method enables the comprehensive identification of bacterial communities and has been used in metagenomic studies of ticks [12–14]. Despite the benefits of the approach, very few studies have used amplicon sequencing for nationwide surveys of tickborne bacterial pathogens.

In this study, we aimed to address this gap by conducting a nationwide survey of bacterial pathogens in ticks collected from cattle and sheep across Kyrgyzstan using amplicon NGS for preliminary pathogen detection, followed by species identification and characterization through PCR and Sanger sequencing. Using this strategy, we obtained comprehensive data on the status of tickborne bacterial pathogens in the region. Our findings contribute to a better understanding of the epidemiology of TBDs and are expected to inform future disease-prevention strategies.

## Methods

### Tick sampling

Ticks were collected from cattle and sheep grazing in seven provinces (Batken, Chuy, Issyk-Kul, Jalal-Abad, Naryn, Osh, and Talas) and 27 districts (Batken, Kadamjai, Leilek; Alamudun, Chuiskii, Issyk-Ata, Kemin, Moscow, Panfilov, Sokuluk; Jeti-Oguz, Ton; Nooken, Toguz-Torous, Toktogul; Ak-Talaa, At-Bashi, Emgek-Talaa, Jumgal, Kochkor, Naryn; Aravan, Nookat, Tolman, Uzgen; Bakai-Ata; and Orto-Arik) across Kyrgyzstan between March and July 2022. The collected ticks were preserved in 70% ethanol, and information, such as collection location, host, and number of ticks, was recorded. The samples were transported to the laboratory for species identification and pathogen detection. Ticks were placed in 2.0-mL tubes and stored at −80 °C until DNA extraction.

### Tick species identification

Ticks were identified to species level and developmental stages using morphological characters under a dissecting microscope (MicroOptix MX100, Wiener Neudorf, Austria) as previously described [15]. After genomic DNA extraction, molecular identification of tick species was confirmed by amplifying cytochrome c oxidase I (*COI*) gene sequences (Supplementary Material Table S1). Polymerase chain reaction (PCR) was performed using AccuPower PCR PreMix (Bioneer, Seoul, Republic of Korea). Each PCR mixture contained 1 μL of each oligonucleotide primer (10 pmol/μL), 3 μL of genomic DNA template, and 15 μL of distilled water. Sterile water served as the negative control for each amplification trial. Amplifications were performed using a ProFlex PCR System (Thermo Fisher Scientific), and the amplified products were analyzed via electrophoresis using an automated QIAxcel^®^ system (QIAgen, Hilden, Germany). PCR products for tick species identification were sequenced using the Sanger method to obtain nucleotide sequences, and phylogenetic analysis was subsequently conducted using reference sequences from GenBank.

### Genomic DNA extraction

Tick samples were individually placed in 2.8-mm bead tubes containing 400 μL of phosphate-buffered saline. The ticks were homogenized twice for 30 s at 4 m/s using a Precelly Evolution homogenizer (Bertin Technologies, Bretonneux, France). The homogenates were centrifuged at 12,000*g* for 10 min, and the supernatant was used for DNA extraction using the MagMAX™ DNA Multi-Sample Ultra 2.0 Kit (Applied Biosystems, Waltham, MA, USA) with a KingFisher Flex system (Thermo Fisher Scientific, Waltham, MA, USA), following the manufacturers’ guidelines. The extracted DNA was stored at −20 °C until further analysis.

### Bacterial 16S rDNA amplification

DNA samples from 546 ticks were subjected to *16S rDNA* amplification to detect bacterial tickborne pathogens. The V3–V4 regions of the *16S rRNA* gene were amplified using universal primers (Supplementary Material Table S1). PCR was performed with Axen High-Q PCR Master Mix (Macrogen, Daejeon, Republic of Korea). Each mixture included 1 μL of each oligonucleotide primer (10 pmol/μL), 2 μL of genomic DNA template, 6 μL of distilled water, and 10 μL of High-Q PCR Master Mix (2×). Negative controls with sterile water were included in each amplification trial. Amplicon products were verified by 1% agarose gel electrophoresis and stored at −20 °C until further analysis. 

### Library preparation, sequencing, and NGS data analysis

Libraries were prepared for sequencing by attaching dual indices and Illumina sequencing adapters to *16S rDNA* amplificons using Nextera-compatible indices (Integrated DNA Technologies, Coralville, IA, USA), following the manufacturer’s instructions. Index PCR was performed under the following conditions: initial denaturation at 95 °C for 3 min; eight cycles of 95 °C for 30 s, 55 °C for 30 s, and 72 °C for 30 s; followed by a final extension at 72 °C for 5 min. Distilled water was used as a negative control in each indexing batch. Index PCR products were purified with Illumina purification beads, and library purity and quantity were assessed using a Qubit dsDNA HS Assay Kit (Invitrogen, Waltham, MA, USA). Libraries were denatured with NaOH according to the manufacturer’s protocol (Illumina).

Sequencing was performed using the MiSeq Reagent Kit V3 on an Illumina MiSeq System. Sequence reads were processed using VSEARCH (version 2.15.1) [16] to filter out low-quality reads with maximum expected error rates of one (maxEE: 1) and chimeric sequences. The filtered sequences were clustered into operational taxonomic units (OTUs) at a 97% sequence identity threshold using USEARCH (version 11.0.667) [17]. OTUs were annotated using reference databases downloaded from Greengenes (V13.8) [18] and SILVA 16S (V132) [19].

### Pathogen characterization by polymerase chain reaction

PCR was performed to detect pathogens of ticks. The pathogen with the highest number of reads per sample in the NGS data was selected for PCR confirmation. The gene targets for the tickborne pathogens (*Anaplasma* spp., *Ehrlichia* spp., spotted fever group Rickettsiae, *Coxiella burnetii*, *Francisella tularensis*, and *Bartonella* spp.) are listed in Supplementary Material Table S1.

Nested PCR was performed using 20 μL of AccuPower PCR PreMix (Bioneer, Daejeon, Republic of Korea). Each reaction mixture contained 1 μL of each oligonucleotide primer (10 pmol/μL), 5 μL of genomic DNA template, and 13 μL of distilled water. A second PCR was conducted using 1 μL of the primary PCR product as the template. Positive controls included genomic DNA of *A. phagocytophilum* (provided by the Division of Zoonotic and Vector-Borne Disease Research) and *E. chaffeensis* (provided by the Division of Bacterial Disease, Korea Disease Control and Prevention Agency). Additional controls consisted of genomic DNA from *B. henselae*, *C. burnetii*, *F. tularensis*, and *R. conorii* (Vircell, Granada, Spain). Sterile water was used as the negative control in each amplification trial. PCR amplifications were performed using a ProFlex PCR System (Thermo Fisher Scientific), and the amplified products were analyzed by electrophoresis using an automated QIAxcel^®^ system (QIAgen). 

### Sanger sequencing and phylogenetic analysis

The PCR products from pathogen species detection were sent to a commercial sequencing service (Cosmo Genetech, Seoul, Republic of Korea) and purified using Exonuclease I and alkaline phosphatase. Sanger sequencing was used to create nucleotide sequences on a 3730 XL DNA analyzer (Applied Biosystems, Foster City, CA, USA). The retrieved sequences were reviewed, edited, and assembled manually, resulting in the generation of bidirectional consensus sequences through BioEdit software (version 5.0.9) [20]. Nucleotide BLAST (National Center for Biotechnology Information, NCBI) was used to match the nucleotide sequences with reference sequences in GenBank. A CLUSTAL Omega (version 1.2.1) [21] sequence alignment was produced. For each pathogen species, the representative sequence with 100% identity was selected for phylogenetic analysis. Phylogenetic trees were constructed using the maximum likelihood method implemented in the MEGA software [22], which was also used to determine the best-fit nucleotide substitution model based on the lowest Bayesian information criterion (BIC) value. A bootstrap analysis with 1,000 replicates was performed to assess branch support.

### Statistical analyses

Differences in pathogen infection rates among tick species, collection sites (provinces), and host animals were evaluated using Fisher’s exact tests implemented on the GraphPad QuickCalcs website [23]. A *P*-value of < 0.05 was considered statistically significant.

## Results

### Identification of tick species

**Fig. 1:**
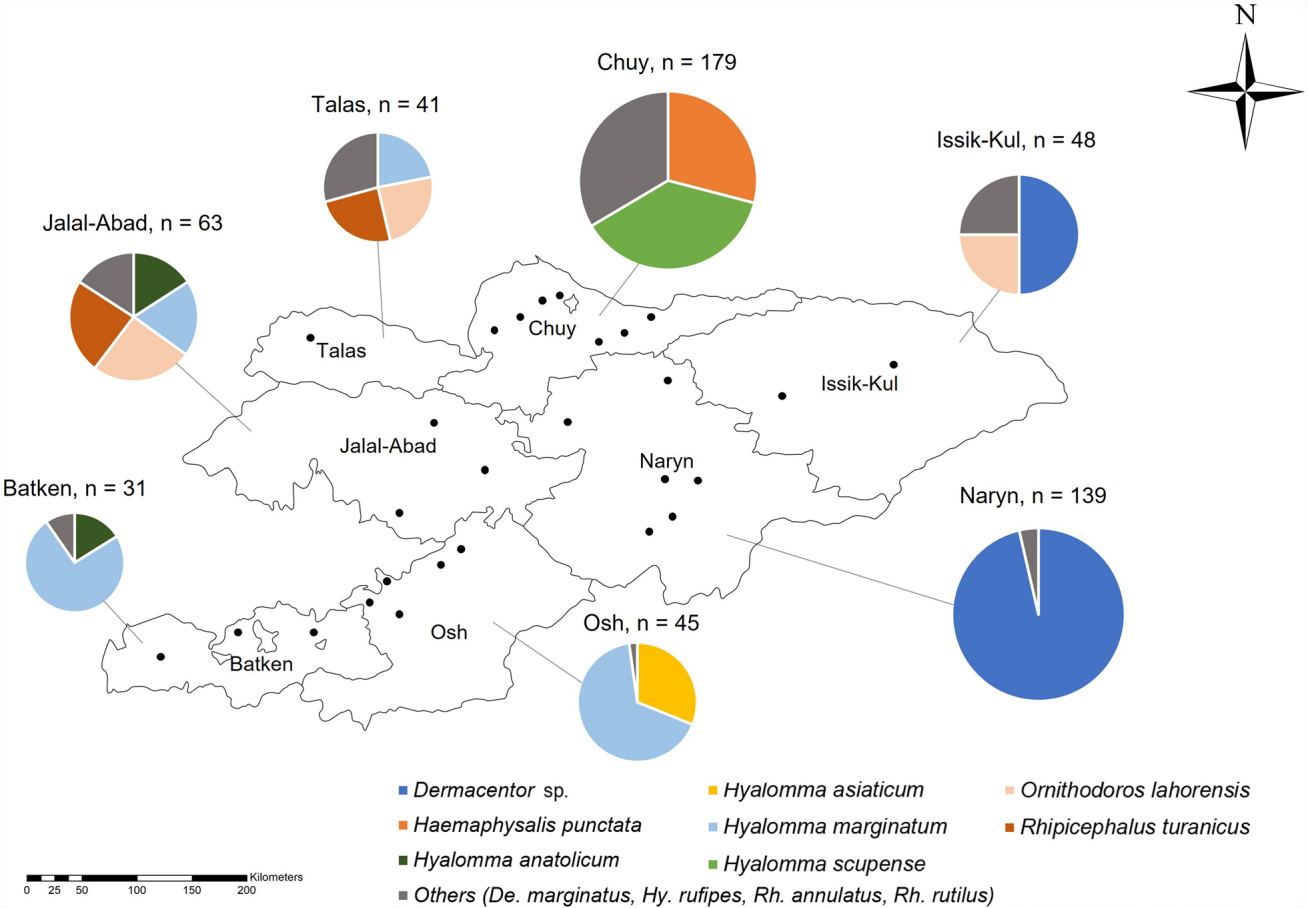
Map showing the composition of tick species in seven provinces. The size of the pie charts corresponds to the number of ticks collected in each region, while the size of each slice within the pie chart represents the proportions of different tick species. The map has been adapted from [24]

**Fig. 2:**
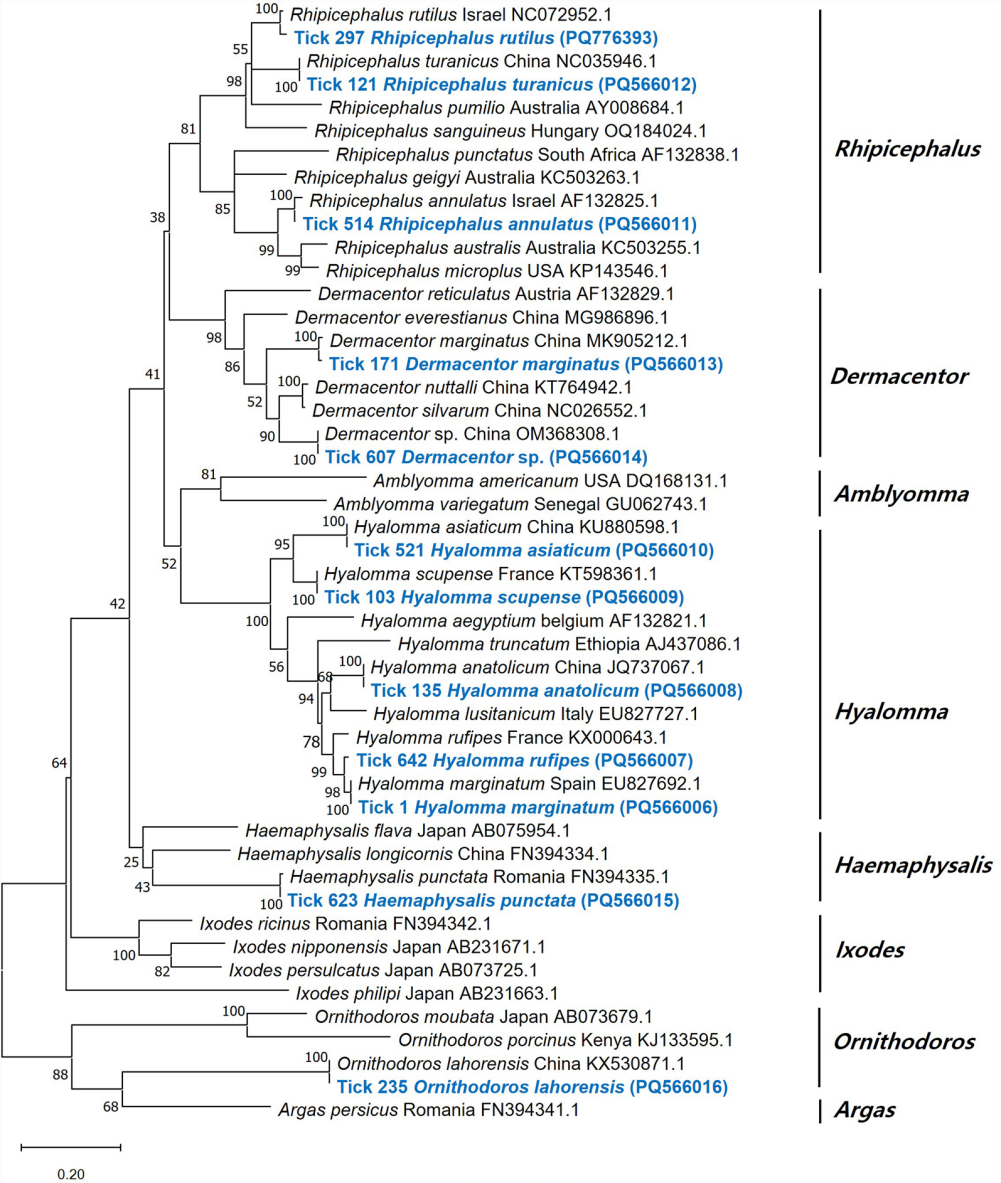
Phylogenetic analysis for tick molecular identification based on partial nucleotide sequences of the *COI* gene fragment (597 bp). The sequences identified in this study are indicated in bold (blue color). The phylogenetic tree was constructed using the maximum likelihood method based on the general time reversible model. Rate variation among sites was modeled using a gamma distribution with invariant sites (G + I). The numbers on the branches indicate bootstrap percentages based on 1,000 replications

A total of 620 ticks were collected from cattle and sheep and morphologically identified, of which, 546 were confirmed by molecular identification, while 74 remained unidentified. The identified ticks belonged to two families (Argasidae and Ixodidae), five genera (*Ornithodoros*, *Dermacentor*, *Haemaphysalis*, *Hyalomma*, and *Rhipicephalus*), and 12 species: *Or. lahorensis*, *Dermacentor spp.*, *De. marginatus*, *Ha. punctata*, *Hy. anatolicum*, *Hy. asiaticum*, *Hy. marginatum*, *Hy. rufipes*, *Hy. scupense*, *Rh. annulatus*, *Rh. rutilus*, and *Rh. turanicus*. The dominant tick species was *Dermacentor* spp. (*n* = 165, 30.2%), followed by *Hy. marginatum* (*n* = 94, 17.2%), *Hy. scupense* (*n* = 73, 13.4%), and *Ha. punctata* (*n* = 64, 11.7%) (Table 1, Fig. 1). Of the 546 nucleotide sequences obtained, 106 unique sequences were selected after excluding those with 100% identity. From these, one representative sequence per species was selected for phylogenetic analysis. A phylogenetic analysis was conducted using representative sequences from each species, confirming that all sequences clustered correctly at the species level (Fig. 2). The sequences shared 98.3–100% identity with previously reported reference sequences (Supplementary Material Table S2) (PQ566006-16, PQ776393). However, several sequences from *Dermacentor* spp. clustered separately from the reference sequence (OM368308.1) in China.

**Table 1:** Tickborne bacterial pathogens detected in tick species infesting cattle and sheep from Kyrgyzstan

Tick genus	Tick species	Hosts	Number of ticks	Number positive for bacterial pathogens (% infection rate)				
				All pathogens	*Anaplasma* spp.	*Ehrlichia* spp.	Spotted fever group Rickettsiae	*Bartonella* spp.
*Dermacentor*	*marginatus*	Cattle	1	0 (0.0)	0 (0.0)	0 (0.0)	0 (0.0)	0 (0.0)
	spp.	Cattle	139	37 (26.6)	0 (0.0)	0 (0.0)	37 (26.6)	0 (0.0)
		Sheep	26	4 (15.4)	1 (3.8)	0 (0.0)	3 (11.5)	0 (0.0)
*Haemaphysalis*	*punctata*	Cattle	34	2 (5.9)	1 (2.9)	0 (0.0)	0 (0.0)	1 (2.9)
		Sheep	30	1 (3.3)	1 (3.3)	0 (0.0)	0 (0.0)	0 (0.0)
*Hyalomma*	*anatolicum*	Cattle	15	0 (0.0)	0 (0.0)	0 (0.0)	0 (0.0)	0 (0.0)
	*asiaticum*	Cattle	11	1 (9.1)	0 (0.0)	0 (0.0)	1 (9.1)	0 (0.0)
		Sheep	3	0 (0.0)	0 (0.0)	0 (0.0)	0 (0.0)	0 (0.0)
	*marginatum*	Cattle	51	2 (3.9)	0 (0.0)	0 (0.0)	2 (3.9)	0 (0.0)
		Sheep	43	4 (9.3)	2 (4.7)	0 (0.0)	2 (4.7)	0 (0.0)
	*rufipes*	Cattle	2	0 (0.0)	0 (0.0)	0 (0.0)	0 (0.0)	0 (0.0)
		Sheep	4	0 (0.0)	0 (0.0)	0 (0.0)	0 (0.0)	0 (0.0)
	*scupense*	Cattle	73	1 (1.4)	0 (0.0)	1 (1.4)	0 (0.0)	0 (0.0)
*Rhipicephalus*	*annulatus*	Cattle	8	0 (0.0)	0 (0.0)	0 (0.0)	0 (0.0)	0 (0.0)
	*rutilus*	Sheep	1	1 (100.0)	1 (100.0)	0 (0.0)	0 (0.0)	0 (0.0)
	*turanicus*	Cattle	24	4 (16.7)	3 (12.5)	0 (0.0)	1 (4.2)	0 (0.0)
		Sheep	31	7 (22.6)	6 (19.4)	0 (0.0)	1 (3.2)	0 (0.0)
*Ornithodoros*	*lahorensis*	Cattle	19	0 (0.0)	0 (0.0)	0 (0.0)	0 (0.0)	0 (0.0)
		Sheep	31	0 (0.0)	0 (0.0)	0 (0.0)	0 (0.0)	0 (0.0)
Total			546	64 (11.7)	15 (2.7)	1 (0.2)	47 (8.6)	1 (0.2)

### Detection of tickborne pathogens

NGS reads ranging from 8 to 15,056 per sample were annotated to multiple bacterial pathogens in ticks, including *Anaplasma*, *Bartonella*, *Coxiella*, *Ehrlichia*, *Francisella*, and *Rickettsia*. These pathogens were selected for confirmatory testing using PCR. In total, 11.7% (64/546) of ticks tested positive for tickborne pathogens. The pathogen rates were as follows: 8.6% for the spotted fever group Rickettsiae, 2.7% for *Anaplasma*, and 0.2% each for *Ehrlichia* and *Bartonella*. By contrast, *Coxiella burnetii* and *Francisella tularensis* could not be confirmed by PCR assays (Tables 1 and 2).

**Table 2:** Tickborne bacterial pathogen detected in ticks infesting cattle and sheep at seven provinces in Kyrgyzstan

Provinces	Number of ticks	Number of bacterial pathogen positive (% infection rate)												
		All pathogens	*Anaplasma* spp.				*Ehrlichia* sp.	Spotted fever group Rickettsiae						*Bartonella* spp.
			*A. bovis*	*A. capra*	*A. ovis*	*Anaplasma* sp.		*R. aeschlimannii*	*R. cornorii*	*R. raoultii*	*R.rickettsii*	*R. sibirica*	*R. slovaca*	*B. bovis*
Cattle														
Batken	23	1 (4.3)	0 (0.0)	0 (0.0)	0 (0.0)	0 (0.0)	0 (0.0)	1 (4.3)	0 (0.0)	0 (0.0)	0 (0.0)	0 (0.0)	0 (0.0)	0 (0.0)
Chuy	133	4 (3.0)	1 (0.8)	0 (0.0)	0 (0.0)	0 (0.0)	1 (0.8)	0 (0.0)	1 (0.8)	0 (0.0)	0 (0.0)	0 (0.0)	0 (0.0)	1 (0.8)
Issyk-Kul	14	0 (0.0)	0 (0.0)	0 (0.0)	0 (0.0)	0 (0.0)	0 (0.0)	0 (0.0)	0 (0.0)	0 (0.0)	0 (0.0)	0 (0.0)	0 (0.0)	0 (0.0)
Jalal-Abad	32	4 (12.5)	0 (0.0)	0 (0.0)	2 (6.3)	1 (3.1)	0 (0.0)	1 (3.1)	0 (0.0)	0 (0.0)	0 (0.0)	0 (0.0)	0 (0.0)	0 (0.0)
Naryn	129	37 (28.7)	0 (0.0)	0 (0.0)	0 (0.0)	0 (0.0)	0 (0.0)	0 (0.0)	1 (0.8)	33 (25.6)	0 (0.0)	1 (0.8)	2 (1.6)	0 (0.0)
Osh	36	1 (2.8)	0 (0.0)	0 (0.0)	0 (0.0)	0 (0.0)	0 (0.0)	1 (2.8)	0 (0.0)	0 (0.0)	0 (0.0)	0 (0.0)	0 (0.0)	0 (0.0)
Talas	10	0 (0.0)	0 (0.0)	0 (0.0)	0 (0.0)	0 (0.0)	0 (0.0)	0 (0.0)	0 (0.0)	0 (0.0)	0 (0.0)	0 (0.0)	0 (0.0)	0 (0.0)
Total	377	47 (12.5)	1 (0.3)	0 (0.0)	2 (0.5)	1 (0.3)	1 (0.3)	3 (0.8)	2 (0.5)	33 (8.8)	0 (0.0)	1 (0.3)	2 (0.5)	1 (0.3)
Sheep														
Batken	8	1 (12.5)	0 (0.0)	0 (0.0)	0 (0.0)	0 (0.0)	0 (0.0)	1 (12.5)	0 (0.0)	0 (0.0)	0 (0.0)	0 (0.0)	0 (0.0)	0 (0.0)
Chuy	46	3 (6.5)	1 (2.2)	1 (2.2)	0 (0.0)	0 (0.0)	0 (0.0)	1 (2.2)	0 (0.0)	0 (0.0)	0 (0.0)	0 (0.0)	0 (0.0)	0 (0.0)
Issyk-Kul	34	2 (5.9)	0 (0.0)	0 (0.0)	0 (0.0)	0 (0.0)	0 (0.0)	0 (0.0)	0 (0.0)	2 (5.9)	0 (0.0)	0 (0.0)	0 (0.0)	0 (0.0)
Jalal-Abad	31	5 (16.1)	0 (0.0)	0 (0.0)	5 (16.1)	0 (0.0)	0 (0.0)	0 (0.0)	0 (0.0)	0 (0.0)	0 (0.0)	0 (0.0)	0 (0.0)	0 (0.0)
Naryn	10	0 (0.0)	0 (0.0)	0 (0.0)	0 (0.0)	0 (0.0)	0 (0.0)	0 (0.0)	0 (0.0)	0 (0.0)	0 (0.0)	0 (0.0)	0 (0.0)	0 (0.0)
Osh	9	0 (0.0)	0 (0.0)	0 (0.0)	0 (0.0)	0 (0.0)	0 (0.0)	0 (0.0)	0 (0.0)	0 (0.0)	0 (0.0)	0 (0.0)	0 (0.0)	0 (0.0)
Talas	31	6 (19.4)	0 (0.0)	0 (0.0)	4 (13.0)	0 (0.0)	0 (0.0)	0 (0.0)	0 (0.0)	0 (0.0)	1 (3.2)	0 (0.0)	1 (3.2)	0 (0.0)
Total	169	17 (10.1)	1 (0.6)	1 (0.6)	9 (5.3)	0 (0.0)	0 (0.0)	2 (1.2)	0 (0.0)	2 (1.2)	1 (0.6)	0 (0.0)	1 (0.6)	0 (0.0)

In terms of tick genus, *Dermacentor* had the highest prevalence of tickborne pathogen infection (*n* = 41/166, 24.7%), followed by *Rhipicephalus* (*n* = 12/64, 18.8%), *Haemaphysalis* (*n* = 3/61, 4.7%), and *Hyalomma* (*n* = 8/202, 4.0%). Infection rates in the *Dermacentor* genus was significantly higher than those in *Haemaphysalis* and *Hyalomma* (Fisher’s exact test, *P* = 0.0003, odds ratio, OR: 6.7, 95% confidence intervals, CI: 0.7–7.7 and *P* < 0.0001, OR: 8.0, 95% CI: 1.1–5.4, respectively). 

The spotted fever group Rickettsiae was detected in all seven provinces, with the Naryn province showing the highest infection rate (26.6%). By contrast, *Anaplasma* spp. were detected in only three provinces (Talas, Jalal-Abad, and Chuy), while *Ehrlichia* spp. and *Bartonella* spp. were detected exclusively in Chuy province (Table 2). Infection rates of tickborne pathogen in Naryn, Talas, and Jalal-Abad were significantly higher than that in Chuy (Fisher’s exact test, *P* < 0.0001, OR: 8.9, 95% CI: 1.1–6.0; *P* = 0.0182, OR: 4.2, 95% CI: 0.6–5.9; *P* = 0.0078, OR: 4.1, 95% CI: 0.7–5.2, respectively). Host-based differences in infection rates were also observed. *Anaplasma* spp. were more frequently detected in ticks collected from sheep (6.5%) than in those from cattle (1.1%). This difference was statistically significant (Fisher’s exact test, *P* = 0.0008, OR: 6.5, 95% CI: 0.7–7.2). By contrast, the spotted fever group Rickettsiae had higher detection rates in cattle ticks (10.9%) than in sheep ticks (3.6%). This difference was statistically significant (*P* = 0.0045, OR: 3.3, 95% CI: 0.7–4.0).

### Phylogenetic analysis of tickborne pathogens

**Fig. 3:**
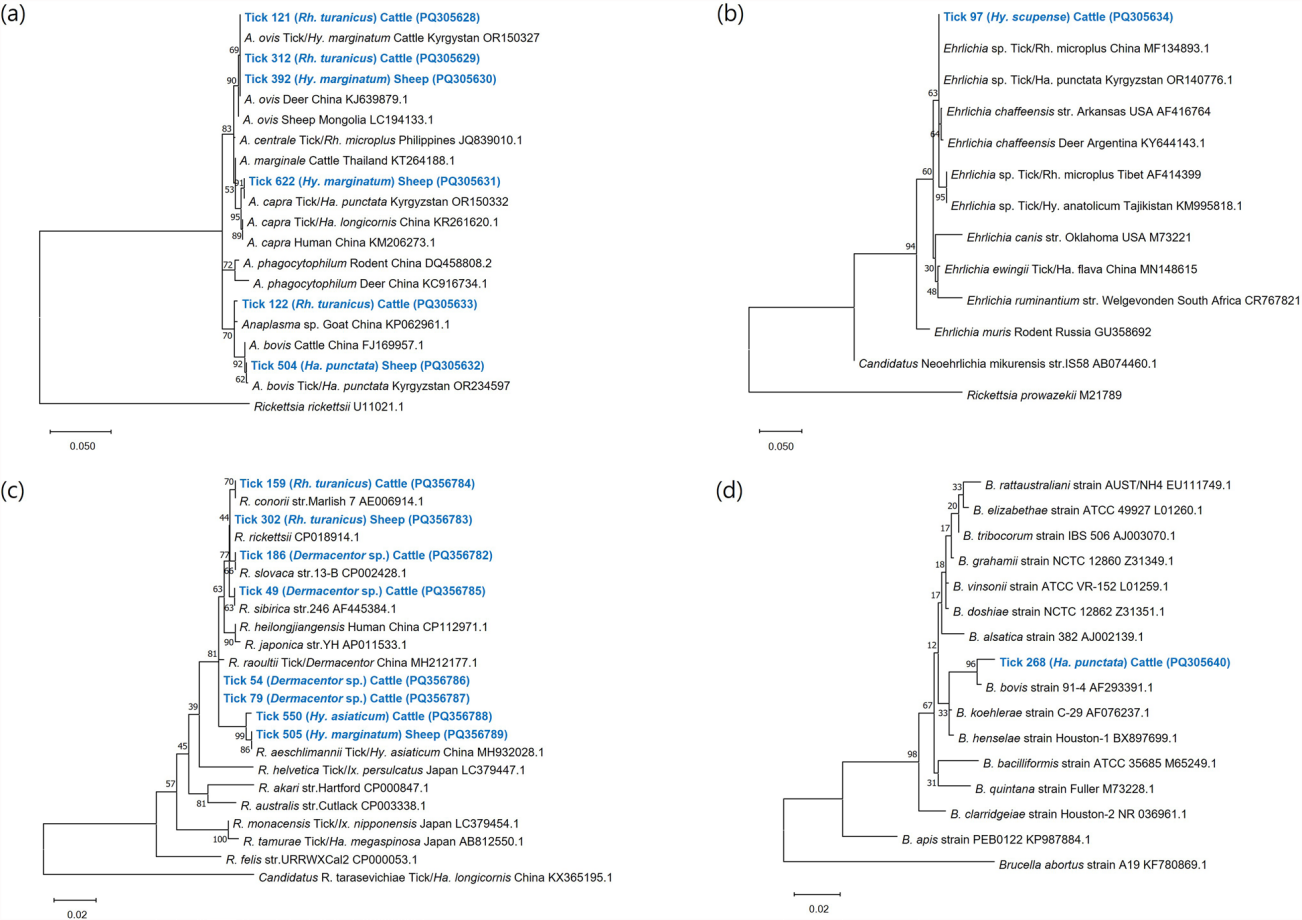
Phylogenetic analysis of tickborne bacterial pathogens (*Anaplasma* spp., *Ehrlichia* spp., spotted fever group Rickettsiae, and *Bartonella* spp.). Phylogenetic analysis of **a** *Anaplasma* spp., **b** *Ehrlichia* spp., and **d** *Bartonella* spp. based on *16S rRNA* gene fragments (878, 385, and 1,020 bp). Phylogenetic analysis of **c** spotted fever group Rickettsiae based on the fragments of the *17 kDa antigen coding* gene (398 bp). The sequences identified in this study are indicated in bold (blue color). The phylogenetic tree was constructed using the maximum likelihood method based on the Kimura 2-parameter model. Rate variation among sites was modeled using a gamma distribution in (**a**–c), and a gamma distribution with a proportion of invariant sites (G + I) in (**d**). The numbers on the branches indicate bootstrap percentages based on 1,000 replications

A phylogenetic analysis based on the *16S rRNA* gene sequences targeting *Anaplasma* identified 11 ticks carrying *A. ovis*, two carrying *A. bovis*, one carrying *A. capra*, and one carrying an unclassified *Anaplasma* sp. (PQ305628–PQ305633). These sequences shared 99.5–100% identity with previously reported sequences from *Hy. marginatum* and *Ha. punctata* ticks in Kyrgyzstan (OR150327, OR150332, and PQ305632) and from deer and goats in China (KJ639879.1 and KP062961.1) (Fig. 3a) (Supplementary Material Table S3). For *Ehrlichia*, sequences (PQ305634) from *Hy. scupense* ticks exhibited 100% nucleotide identity with *Ehrlichia* sp. detected in *Ha. punctata* and *R. microplus* ticks in Kyrgyzstan and China (OR140776.1, MF134893.1) (Fig. 3b). A phylogenetic analysis based on the *17kD antigen coding *gene in 47 ticks positive for the spotted fever group Rickettsiae revealed six species: *R. aeschlimannii*, *R. conorii*, *R. raoultii*, *R. rickettsia*, *R. sibirica*, and *R. slovaca* (PQ356782–PQ356789) (Fig. 3c). The *R. raoultii* sequences obtained in this study showed 99.5–99.7% identity with a previously reported sequence from *Dermacentor* sp. in China (MH212177.1). Similarly, *R. aeschlimannii* sequences showed 99.5–100% identity with sequences from *Hy. asiaticum* ticks in China (MH932028.1). The sequences identified as *R. conorii*, *R. rickettsii*, *R. sibirica*, and *R. slovaca* exhibited 99.7%, 100%, 100%, and 100% identity, respectively, with reference sequences from the same clade (AE006914.1, CP018914.1, AF445384.1, and CP002428.1). Additionally, the *Bartonella* sequence (PQ305640) obtained from *Ha. punctata* ticks collected from cattle was 99.1% identical to the *B. bovis* strain 91-4 (AF293391.1) (Fig. 3d).

## Discussion

Pathogen detection in ticks using targeted approaches, such as single or multiplex PCR, primer extension, or bait probe enrichment, is limited by the need to target predefined pathogens, often requiring multiple individual tests. Additionally, these methods may fail to detect rare pathogens owing to primer mismatches with genetically divergent strains [25]. NGS overcomes these limitations by enabling the broad detection of bacterial pathogens in a single analysis. Conventional assays paired with Sanger sequencing are still necessary for species confirmation, making this combination a time-efficient and effective strategy [14].

Tick samples are often damaged during collection and storage, complicating morphological identification. Only experienced taxonomists can reliably distinguish species on the basis of external features, which poses challenges for diagnosis and pathogen surveillance. DNA-based methods, particularly those targeting mitochondrial DNA (mtDNA), provide a more accurate alternative by assessing genetic divergence between species. In particular, the cytochrome c oxidase I (*COI*) gene is widely recognized as an effective molecular marker for species identification [26]. In this study, ticks were identified using the *COI* marker and classified into 12 species from five genera and two families. The most prevalent species was *Dermacentor* spp. (*n* = 165, 30.2%), followed by *Hy. marginatum* (*n* = 94, 17.2%) and *Hy. scupense* (*n* = 73, 13.4%). Previous studies have reported 14 tick species in Kyrgyzstan belonging to the genera *Dermacentor* and *Argas*, *Hyalomma*, *Haemaphysalis*, *Rhipicephalus*, *Ixodes*, and *Ornithodoros* [27, 28]. In the Chuy region, *Ar. persicus* (26.5%), *Ha. punctata* (18.0%), and *Dermacentor* spp. (16.0%) were identified as dominant species infesting livestock [10]. This study presents the first report of *Hy. rufipes* in Kyrgyzstan. However, previous studies have shown that using a single gene for species identification in ticks may have limitations [29–31]. In this study, ticks belonging to the *Dermacentor* genus formed a distinct clade independent of previously reported species. In addition, morphological identification did not allow for the species-level classification of *Dermacentor* ticks. This limitation may have influenced the comparison of pathogen infection rates among tick species and could have affected the statistically significant result indicating that *Dermacentor* ticks had the highest infection rate. Therefore, accurate tick identification would require not only morphological identification but also further molecular investigation of additional molecular markers, such as *16S rRNA*, *ITS2*, and *12S rRNA*. 

The spotted fever group Rickettsiae was the most prevalent bacterial pathogen identified in ticks with an infection rate of 8.6%. Previous studies in Kyrgyzstan have not investigated this pathogen group since *R. sibirica* was reported in rodents in 1963 [32]. This study reports the first detection on the spotted fever group Rickettsiae in ticks from Kyrgyzstan, with positive samples identified from *Dermacentor* sp., *Rh. turanicus*, *Hy. asiaticum*, and *Hy. marginatum*. Of the 47 positive samples, 33 were identified as *R. raoultii*, detected exclusively in the Naryn and Issyk-Kul provinces, which border the Xinjiang Uygur Autonomous Region (XUAR) of northwestern China. A 2020 survey in XUAR found a *R. raoultii* positivity rate of 36.8% in *De. nuttalli*, *De. marginatus*, and *Hy. anatolicum* ticks. Another study in the region reported positivity rates of *R. raoultii* (12.8%), *R. massiliae* (9.1%), *R. aeschlimannii* (2.6%), and *R. sibirica* (2.6%) in *De. nuttalli*, *De. marginatus*, *Ha. punctata,* and *Rh. turanicus* ticks. *Rickettsia raoultii* is an etiological agent of scalp eschar and neck lymphadenopathy (SENLAT) syndrome, which is transmitted by *De. marginatus* and *De. reticulatus*. Clinical manifestations of this disease include fever, headache, fatigue, rash, scalp eschar, lymphadenopathy, and facial edema. Other species detected in this study are known to cause human diseases, such as spotted fever (*R. aeschlimannii*), Mediterranean spotted fever (*R. conorii*), Rocky Mountain spotted fever (*R. rickettsia*), SENLAT (*R. slovaca*), and Siberian tick typhus (*R. sibirica*) [33].

*Anaplasma* and *Ehrlichia* are obligate intracellular bacteria belonging to the family *Anaplasmataceae* and are transmitted by infected ticks. These pathogens present similar clinical symptoms, including fever, headache, leukopenia, thrombocytopenia, and elevated liver enzyme levels, and are responsible for zoonotic diseases, such as anaplasmosis and ehrlichiosis. The genera *Ixodes*, *Dermacentor*, *Rhipicephalus*, and *Amblyomma* are the primary vectors of *Anaplasma*, while *Amblyomma* and *Rhipicephalus* are the main vectors of *Ehrlichia* [34].

Based on a *16S rRNA* gene sequence analysis, *Anaplasma* species identified in this study included *A. ovis*, *A. bovis*, *A. capra*, and an unclassified *Anaplasma* sp. These species were detected in ticks belonging to *Rh. turanicus*, *Rh. rutilus*, *Dermacentor* sp., *Ha. punctata,* and *Hy. marginatum*. In a previous study conducted in Chuy, Kyrgyzstan, *A. bovis* (8.9%), *Anaplasma* sp. (4.0%), *A. ovis* (1.0%), and *A. capra* (0.4%) were detected in *R. turanicus*, *R. annulatus*, *Dermacentor* sp., *Ha. punctata*, *Hy. marginatum*, *Hy. scupense,* and *Argas persicus* ticks [10]. Another study reported the presence of *A. centrale*, *A. capra*, and *A. phagocytophilum*-like bacteria only in Chuy region after examining cattle in Chuy, Talas, Jalal-Abad, Naryn, and Issyk-Kul in Kyrgyzstan [7]. However, in this study, *Anaplasma* infection rates were highest in Jalal-Abad province (12%), followed by Talas (9%) and Chuy (1%). Notably, *A. ovis* showed the highest prevalence among *Anaplasma* species, followed by *A. bovis* and *A. capra*. While *A. ovis* and *A. capra* are zoonotic pathogens, *A. bovis* primarily causes disease in animals [35]. These findings are consistent with a study from the XUAR region of China, where *A. ovis* was detected in 22.4% of ticks including *Hy. anatolicum*, *D. nuttalli,* and *D. marginatus* [36]. Another study found that the positivity rate of *A. ovis* in sheep (63.8%) was much higher than that of *A. bovis* in cattle (5.1%) [37], which aligns with the results of the current study, where *Anaplasma* was detected more frequently in ticks from sheep than in those from cattle.

In this study, *Ehrlichia* sp. was identified in *Hy. scupense*, with sequences showing 100% identity to those of *Ehrlichia* detected in *Ha. punctata* ticks in Kyrgyzstan and *Rh. microplus* ticks in China. In a previous study conducted in Chuy, *E. chaffeensis* and *Ehrlichia* sp. were detected in *Ha. punctata* ticks [10]. However, to date, there are no reported cases of human ehrlichiosis in Kyrgyzstan.

*Bartonella* is a zoonotic pathogen that is transmitted from mammals to humans by various insect vectors, including sandflies, cat fleas, and human body lice. There is emerging evidence that ticks, red ants, and spiders may also transmit *Bartonella*. The most common clinical manifestations in humans include lymphadenopathy, bacillary angiomatosis, peliosis, uveitis, endocarditis, and prolonged fever [38]. In this study, *B. bovis* was identified in *Ha. punctata* ticks collected from cattle. To our knowledge, this is the first report of the *Bartonella* genus in ticks from Kyrgyzstan. *B. bovis* has been previously implicated as the etiological agent in a human case of bartonellosis [39]. Additionally, prolonged bacteremia in healthy animals can lead to *B. bovis*-associated endocarditis in elderly cows [40].

This study had several limitations. In this study, ticks were collected from seven regions across Kyrgyzstan, with 31–139 ticks collected from each region. A limitation of the study is that the number of specimens collected is insufficient to fully represent the tick species distribution and pathogen positivity rates for that region. As the collection period spanned from March to July, there is a possibility that specific developmental stages (adults and nymphs) were sampled, which may not reflect seasonal variations in species composition and pathogen infection rates. Furthermore, only the most abundant bacterial genus in each tick sample was determined, limiting analyses of coinfections. Additionally, the detection of microbial DNA does not confirm the presence of viable organisms, as DNA from a recent blood meal may be present [41]. Future research should focus on improving detection methods to assess pathogen viability and investigate coinfection dynamics.

This study confirmed the presence of bacterial pathogens such as *Anaplasma*, *Ehrlichia,* Spotted fever group Rickettsiae, and *Bartonella* in ticks in Kyrgyzstan. Exposure to infected vector ticks remains a key risk factor for acquiring tickborne diseases (TBDs). Strengthening national surveillance systems for ticks and tickborne pathogens can enhance our understanding of regional variations in TBD risk factors. Accordingly, the findings of this study may contribute valuable insights for the establishment of effective surveillance strategies and the formulation of public health policies targeting TBDs in Kyrgyzstan, ultimately contributing to the reduction of tickborne disease incidence in the country.

## Conclusions

This is the first nationwide study of bacterial pathogens in ticks in Kyrgyzstan using NGS, with spotted fever group Rickettsiae and *Bartonella* reported in ticks for the first time in the country. The detection of pathogens causing diseases such as spotted fever, anaplasmosis, ehrlichiosis, and bartonellosis in ticks infesting cattle and sheep suggests a potential risk of transmission to both animals and humans. Therefore, the continuous monitoring of tickborne pathogens is essential to mitigate the risk of emerging tickborne diseases in Kyrgyzstan. This study contributes to a better understanding of the epidemiology of these pathogens and supports the use of amplicon NGS, followed by pathogen-specific assays, as a time-effective strategy for screening arthropod vectors.

## Supplementary Information

Supplementary Material 1. Table S1. Target genes and primer sequences used for conventional PCR of tickborne bacterial pathogens in ticks infesting cattle and sheep from Kyrgyzstan. Table S2. Comparison of *COI* gene DNA Sequence similarities between ticks in this study and reference sequences in GenBank. Table S3. Comparison of DNA sequence similarities between pathogens detected in ticks and reference sequences in GenBank.

## Data Availability

No datasets were generated or analyzed during the current study.

## Abbreviations

NGS: Next-generation sequencing
PCR: Polymerase chain reaction
COI: Cytochrome c oxidase I
16S rRNA: 16S ribosomal ribonucleic acid
TBDs: Tickborne diseases
