# Supplementary material for: Identification of tickborne pathogens in cattle and sheep ticks from Kyrgyzstan using next-generation sequencing
Source: Parasit Vectors. 2025 Jul 22;18:292. doi: 10.1186/s13071-025-06919-4 (PMC12285077; doi:10.1186/s13071-025-06919-4)
Supplement: Supplementary file 1 — Supplementary Material 1. Table S1. Target genes and primer sequences used for conventional PCR of tickborne bacterial pathogens in ticks infesting cattle and sheep from Kyrgyzstan. Table S2. Comparison of COI gene DNA Sequence similarities between ticks in this study and reference sequences in GenBank. Table S3. Comparison of DNA sequence similarities between pathogens detected in ticks and reference sequences in GenBank. [file 13071_2025_6919_MOESM1_ESM.docx]

**Supplementary material**

Table S1. Target genes and primer sequences used for conventional PCR of tick-borne bacterial pathogens in ticks infesting cattle and sheep from Kyrgyzstan.

| Pathogen | Target gene | Primer name | Nucleotide sequence (5’-3’) | Product size (bp) | PCR conditions | Reference |
| --- | --- | --- | --- | --- | --- | --- |
| Tick species | COⅠ | LCO1490 | GGTCAACAAATCATAAAGATATTGG | 710 | 95 °C 5 m; 35 cycles: 95 °C 1 m, 40 ℃ 1 m, 72 ℃ 30 s; 72 ℃ 10 m | [42] |
|  |  | HC02198 | TAAACTTCAGGGTGACCAAAAAATCA |  |  |  |
| Bacterial pathogens | 16S rRNA V3-V4 | Bakt 341F | CCTACGGGNGGCWGCAG | 465 | 95 °C 3 m; 35 cycles: 98 °C 20 s, 55 °C 30 s, 72 °C 30 s; 72 °C 5 m | [43, 44] |
|  |  | Bakt 805R | GACTACHVGGGTATCTAATCC |  |  |  |
| *Anaplasma* spp. | 16S rRNA | AE1-F | AAGCTTAACACATGCAAGTCGAA | 1406 | 94 °C 5 m; 40 cycles: 94 °C 1m, 59 ℃ 1 m, 72 ℃ 90 s; 72 ℃ 10 m | [45] |
|  |  | AE1-R | AGTCACTGACCCAACCTTAAATG |  |  |  |
|  |  | EE3 | GTCGAACGGATTATTCTTTATAGCTTGC | 926 | 94 ℃ 5 m; 35 cycles: 94 ℃ 30 s, 50 ℃ 30 s, 72 ℃ 1 m; 72 ℃ 10 m |  |
|  |  | EE4 | CCCTTCCGTTAAGAAGGATCTAATCTCC |  |  |  |
| *Ehrlichia* spp. | 16S rRNA | AE1-F | AAGCTTAACACATGCAAGTCGAA | 1406 | 94 ℃ 5 m; 40 cycles: 94 ℃ 1m, 59 ℃ 1 m, 72 ℃ 90 s; 72 ℃ 10 m | [46] |
|  |  | AE1-R | AGTCACTGACCCAACCTTAAATG |  |  |  |
|  |  | HE1 | CAATTGCTTATAACCTTTTGGTTATAAAT | 390 | 94 ℃ 3 m; 3 cycles: 94 ℃ 1 m, 55 ℃ 2 m, 72 ℃ 90 s; 92 ℃ 1 m; 37 cycles: 92 ℃ 1 m, 55 ℃ 2 m, 72 ℃ 1 m; 72 ℃ 10 m | [47] |
|  |  | HE3 | TATAGGTACCGTCATTATCTTCCCTAT |  |  |  |
| Spotted fever group Rickettsiae | 17 kDa antigen | Rr17k.1p | TTTACAAAATTCTAAAAACCAT | 539 | 95 ℃ 5 m; 35 cycles: 95 ℃ 30 s, 57 ℃ 1 m, 72 ℃ 2 m; 72 ℃ 5 m | [48] |
|  |  | Rr17k.539n | TCAATTCACAACTTGCCATT |  |  |  |
|  |  | Rr17k.90p | GCTCTTGCAACTTCTATGTT | 450 | 95 ℃ 5 m; 35 cycles: 95 ℃ 30 s, 57 ℃ 1 m, 72 ℃ 2 m; 72 ℃ 5 m |  |
|  |  | Rr17k.539n | TCAATTCACAACTTGCCATT |  |  |  |
| *Coxiella burnetii* | IS1111 | IS-F | CAGAGCCACCGTATGAATCAGCTT | 959 | 95 ℃ 4 m; 32 cycles: 95 ℃ 55 s, 5 cycles: 66 ℃ - 63 ℃ (touchdown) 55 s, 72 ℃ 55 s; 72 ℃ 7 m | [49] |
|  |  | IIS-R | TCGGACGTTTATGGGGATGGGTAT |  |  |  |
|  |  | ISNF | CACATTGCCGCGTTTACTAATCCC | 421 | 95 ℃ 4 m; 30 cycles: 95 ℃ 15 s, 66 ℃ 15 s, 72 ℃ 15 s; 72 ℃ 7 m |  |
|  |  | ISNR | CACGGCGCTGATCAATGAGATTC |  |  |  |
| *Francisella tularensis* | fopA | FNA8L-F | CGAGGAGTCTCAATGTACTAAGGTTTGCCC | 900 | 95 ℃ 3 m; 35 cycles: 95 ℃ 15 s, 55 ℃ 15 s, 72 ℃ 30 S; 72 ℃ 10 m | [50] |
|  |  | FNB2L-R | CACCATTATCCTGGATATTACCAGTGTCAT |  |  |  |
|  |  | FNA7L-F | CTTGAGTCTTATGTTTCGGCATGTGAATAG | 409 | 95 ℃ 3 m; 35 cycles: 95 ℃ 15 s, 55 ℃ 15 s, 72 ℃ 30 S; 72 ℃ 10 m |  |
|  |  | FNB1L-R | CCAACTAATTGGTTGTACTGTACAGCGAAG |  |  |  |
| *Bartonella* spp. | 16S rRNA | A-proteo | AGAGTTTGATMCTGGCTCAGA | 1210 | 94 ℃ 5 m; 35 cycles: 94 ℃ 1m, 62 ℃ 1 m, 72 ℃ 1 m; 72 ℃ 5 m | [51] |
|  |  | r-Alpha-sh | GTAGCACGTGTGTAGCCCA |  |  |  |
|  |  | Bart | CACTCTTTTAGAGTGAGCGGCAA | 990 | 94 ℃ 5 m; 35 cycles: 94 ℃ 1m, 65 ℃ 1 m, 72 ℃ 1 m; 72 ℃ 5 m |  |
|  |  | r-BH | CCCCCTAGAGTGCCCAACCA |  |  |  |

Table S2. Comparison of COI gene DNA Sequence similarities between ticks in this study and reference sequences in GenBank.

| Tick | Tick species GenBank match (Accession number) | Length | Identity |
| --- | --- | --- | --- |
| Tick 135 | *Hyalomma anatolicum* (JQ737067.1) | 597 bp | 100% |
| Tick 642 | *Hyalomma rufipes* (KX000643.1) | 597 bp | 96.1% |
| Tick 1 | *Hyalomma marginatum* (EU827692.1) | 597 bp | 100% |
| Tick 521 | *Hyalomma asiaticum* (KU880598.1) | 597 bp | 100% |
| Tick 103 | *Hyalomma scupense* (KT598361.1) | 597 bp | 100% |
| Tick 297 | *Rhipicephalus rutilus* (NC072952.1) | 597 bp | 98.5% |
|  | *Rhipicephalus turanicus* (NC035946.1) | 597 bp | 92.1% |
|  | *Rhipicephalus sanguineus* (OQ184024.1) | 597 bp | 90.8% |
| Tick 121 | *Rhipicephalus turanicus* (NC035946.1) | 597 bp | 100% |
| Tick 514 | *Rhipicephalus annulatus* (AF132825.1) | 597 bp | 98.7% |
| Tick 171 | *Dermacentor marginatus* (MK905212.1) | 597 bp | 98.8% |
| Tick 607 | *Dermacentor sp.* (OM368308.1) | 597 bp | 100% |
| Tick 623 | *Haemaphysalis punctata* (FN394335.1) | 597 bp | 99.5% |
| Tick 235 | *Ornithodoros lahorensis* (KX530871.1) | 597 bp | 100% |

Table S3. Comparison of DNA sequence similarities between pathogens detected in ticks and reference sequences in GenBank.

| Tick (Species) | Pathogen GenBank Match  (Accession Number) | Gene | Length | Identity |
| --- | --- | --- | --- | --- |
| Tick 392 (*Hyalomma marginatum*) | *Anaplasma ovis* (KJ639879.1) | 16S rRNA | 878 bp | 100% |
| Tick 312 (*Rhipicephalus turanicus*) |  |  |  | 100% |
| Tick 121 (*Rhipicephalus turanicus*) |  |  |  | 99.9% |
| Tick 622 (*Hyalomma marginatum*) | *Anaplasma capra* (OR150332) | 16S rRNA | 878 bp | 100% |
| Tick 122 (*Rhipicephalus turanicus*) | *Anaplasma* sp. (KP062961.1) | 16S rRNA | 878 bp | 99.5% |
| Tick 504 (*Haemaphysalis punctata*) | *Anaplasma bovis* (FJ169957.1) | 16S rRNA | 878 bp | 99.9% |
| Tick 97 (*Hyalomma scupense*) | *Ehrlichia* sp. (MF134893.1) | 16S rRNA | 385 bp | 100% |
| Tick 186 (*Dermacentor* sp.) | *Rickettsia slovaca* (CP002428.1) | 17 kDa antigen | 398 bp | 100% |
| Tick 302 (*Rhipicephalus turanicus*) | *Rickettsia rickettsii* (CP018914.1) | 17 kDa antigen | 398 bp | 100% |
| Tick 159 (*Rhipicephalus turanicus*) | *Rickettsia conorii* (AE006914.1) | 17 kDa antigen | 398 bp | 99.7% |
| Tick 49 (*Dermacentor* sp.) | *Rickettsia sibirica* (AF445384.1) | 17 kDa antigen | 398 bp | 100% |
| Tick 54 (*Dermacentor* sp.) | *Rickettsia raoultii* (MH212177.1) | 17 kDa antigen | 398 bp | 99.5% |
| Tick 79 (*Dermacentor* sp.) |  |  |  | 99.7% |
| Tick 550 (*Hyalomma asiaticum*) | *Rickettsia aeschlimannii* (MH932028.1) | 17 kDa antigen | 398 bp | 99.5% |
| Tick 505 (*Hyalomma marginatum*) |  |  |  | 100% |
| Tick 268 (*Haemaphysalis punctata*) | *Bartonella bovis* (AF293391.1) | 16S rRNA | 1,020 bp | 99.1% |
